# Engineering integrative vectors based on phage site-specific recombination mechanism for *Lactococcus lactis*

**DOI:** 10.1186/s12896-019-0575-x

**Published:** 2019-11-27

**Authors:** Innanurdiani Koko, Adelene Ai-Lian Song, Mas Jaffri Masarudin, Raha Abdul Rahim

**Affiliations:** 1 Department of Cell and Molecular Biology, Faculty of Biotechnology and Biomolecular Sciences, University Putra Malaysia, 43400 UPM, Serdang, Selangor Malaysia; 2 Department of Microbiology, Faculty of Biotechnology and Biomolecular Sciences, University Putra Malaysia, 43400 UPM, Serdang, Selangor Malaysia; 3 Institute of Bioscience, University Putra Malaysia, 43400 UPM Serdang, Selangor Malaysia; 4 Chancellory, Universiti Teknikal Malaysia, 76100 Durian Tunggal, Melaka, Malaysia

**Keywords:** *Lactococcus lactis*, Integrative vector, Secretion, Surface display, Signal peptide

## Abstract

**Background:**

Site-specific integration system allows foreign DNA to be integrated into the specific site of the host genome, enabling stable expression of heterologous protein. In this study, integrative vectors for secretion and surface display of proteins were constructed based on a lactococcal phage TP901–1 integrating system.

**Results:**

The constructed integration system comprises of a lactococcal promoter (*P*_nisA_ or *P*_170_), phage attachment site (attP) from bacteriophage TP901–1, a signal peptide (USP45 or SPK1) for translocation of the target protein, and a PrtP_344_ anchor domain in the case of the integrative vectors for surface display. There were eight successfully constructed integrative vectors with each having a different combination of promoter and signal peptide; pS1, pS2, pS3 and pS4 for secretion, and pSD1, pSD2, pSD3 and pSD4 for surface display of desired protein. The integration of the vectors into the host genome was assisted by a helper vector harbouring the integrase gene. A nuclease gene was used as a reporter and was successfully integrated into the *L. lactis* genome and Nuc was secreted or displayed as expected. The signal peptide SPK1 was observed to be superior to USP45-LEISSTCDA fusion in the secretion of Nuc. As for the surface display integrative vector, all systems developed were comparable with the exception of the combination of *P*_170_ promoter with USP45 signal peptide which gave very low signals in whole cell ELISA.

**Conclusion:**

The engineered synthetic integrative vectors have the potential to be used for secretion or surface display of heterologous protein production in lactococcal expression system for research or industrial purposes, especially in live vaccine delivery.

## Background

*Lactococcus lactis*, a lactic acid bacteria (LAB) that has been conventionally known as a starter culture in food fermentations such as cheese and yoghurt, was granted with the Generally Recognized as Safe (GRAS) status by the US FDA. It is the most well studied LAB strain due to its well characterised genome which allows researchers to manipulate this bacterium for desired applications. *L. lactis* is also known as the model LAB and a promising bacteria in recombinant protein technology that has been extensively used as cell factory for the production of industrially important protein such as enzymes, compounds, antigens, allergens, and cytokines [1–5].

Due to its extensive applications, numerous cloning and expression vectors have been constructed for this bacterium. However, the instability of the replicating plasmid DNA is a drawback where it can be lost under non-selective conditions [6]. Plasmid copy numbers often varies with growth, resulting in different expression levels of the genes they carry [7]. Besides, over expression of heterologous protein causes metabolic load which burdens the host and affect the protein integrity [8]. Due to these limitations, constructions of more stable expression systems were attempted through genomic integration of integrative vectors harbouring the desired gene in *L. lactis*. DNA integrating system has been demonstrated in other LAB such as *Lactobacillus* for vaccine construction, for instance, the expression of surface anchored llama heavy chain antibody against rotavirus [9–12] and other Gram-positive bacteria such as *Bacillus* sp. using spore surface display system to express target protein on the outer surface of bacteria [13]. The integrating system has also been long developed for surface display of target protein for vaccine production in mycobacteria [14, 15] and other applications [16–18]*.* The most well characterised, easy manipulated bacteria, *Escherichia coli* has a long lists of integrating systems developed to express various kinds of recombinant protein for numerous applications [19–24].

As reported by Leenhouts et al., (1990), chromosomal integration in *L. lactis* mediated by replacement recombination and Campbell-like mechanism [25] resulted in unstable integration and the integrated DNA fragment were gradually lost under non-selective condition. The instability of the integrated plasmid was due to its replicative activity and the direct repeats flanking the inserted DNA sequences resulted from the Campbell-like integration permitted intra-molecular homologous recombination by the host system. These conditions caused the integrated plasmid to be cleaved out from the genome, losing the integrated plasmid [26–28].

A site-specific recombination system for *L. lactis* has been developed to generate stable, single-copy chromosomal integration based on the lactococcal temperate bacteriophage TP901–1. The system requires phage-encoded elements, which are the phage attachment site, *attP* and an integrase protein which facilitates recombination between the *attP* site and the bacterial attachment site, *attB* on the host genomic DNA at a high frequency. For the recombination to occur, integrase recognises a short sequence of the site (43 bps) which makes the system applicable in numerous *L. lactis* strains [29–34]. Through utilisation of this established integration system, we developed a variety of integrative vectors for secretion and surface display of targeted proteins and compared their efficiency using nuclease as a reporter.

*L. lactis* possess only one exported housekeeping protease (HtrA), and secrete only one major extracellular protein, USP45, thus minimizing protein degradation by the extracellular proteolytic system, simplifying downstream purification [35]. Secretion is preferable in protein delivery when the target location is the digestive tract of humans or animals, because it facilitates interaction between the expressed protein and its target [36]. On the other hand, surface display enables expression of recombinant protein on the surface of bacteria for various applications such as live vaccine development, antibody production, peptide library screening, and production of whole-cell biocatalysis and bioadsorbents for environmental applications [37].

In this report, we describe the construction of a single-copy integration vector for *L. lactis* based on the TP901–1 site-specific recombination. The integrative vectors were suicide vectors for *L. lactis* host, designed to replicate in *Escherichia coli* for easy DNA manipulations. When it is transformed into the *L. lactis*, the vector integrates into the *attB* site of the genome and the expressed target protein is either secreted or surface displayed on the outer part of the cells. Different combinations of promoters and signal peptides were used and protein expression and secretion efficiency were compared between systems.

## Results

### Helper plasmid (pNZint)

**Fig. 1:**
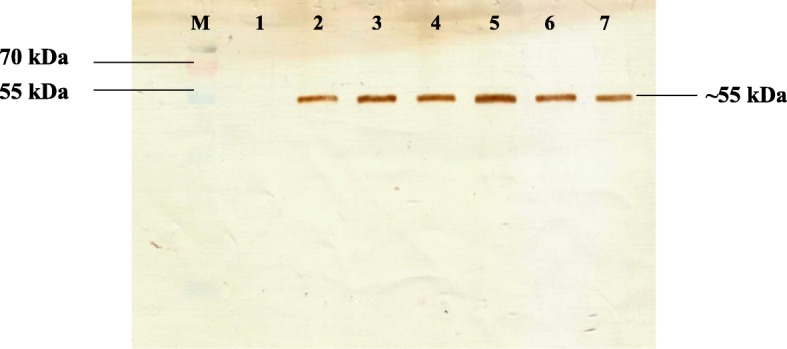
Western blot showing time optimization for the expression of integrase protein. Lane M) Pageruler prestained protein ladder, 1–7) protein sample at 0 h, 2 h, 4 h, 6 h, 8 h, 10 h and 12 h respectively

*L. lactis* harbouring the helper plasmid, pNZint was analysed for the expression of integrase protein as integration of the foreign DNA is only possible with co-expression of the integrase gene. The integrase protein was tagged with 6 histidine residues to allow detection via Western blotting. Protein expression was analysed for 12 h post-induction (20 ng/ml nisin) with 2 h intervals. The expected size of the desired protein was 55.6 kDa where expression of integrase was observed from 2 h onwards (Fig. 1).

### Vector constructions and integration

A total of eight vectors, pS1–4 and pSD1–4 were successfully constructed as listed in Table 1. The constructed secretion and surface display integrative vectors were subsequently cloned with *nuc* gene, thereby named pS1–4nuc and pSD1–4nuc. The integrative vectors harbouring *nuc* were then transformed into *L. lactis* NZ9000 harbouring pNZint, and the positive integrants which would obtain erythromycin resistance were then tested for their stability to stay integrated in the attB site of the genome. After 100 generations grown in non-selective conditions, all random colonies chosen which were sub-cultured on to erythromycin plates had positive growth, indicating that the integrated plasmid was still inside and has not been excised. In addition, PCR using the attB flanking primers (F-attB/R-attB) on genomic DNA extracted from the integrants further confirmed presence of the integrated plasmid.

**Table 1:** Summary of secretion and surface display integrative vector components

Plasmid constructs	Size (bps)	Promoter	Signal peptide	Propeptide	LPXTG anchor domain	Function
pS1	3111	*P*_170_	USP45	LEISSTCDA	–	Secretion
pS2	3072	*P*_170_	SPK1	–	–	Secretion
pS3	3144	*P*_nisA_	USP45	LEISSTCDA	–	Secretion
pS4	3086	*P*_nisA_	SPK1	–	–	Secretion
pSD1	4152	*P*_170_	USP45	LEISSTCDA	PrtP_344_	Surface display
pSD2	4107	*P*_170_	SPK1	–	PrtP_344_	Surface display
pSD3	4185	*P*_nisA_	USP45	LEISSTCDA	PrtP_344_	Surface display
pSD4	4146	*P*_nisA_	SPK1	–	PrtP_344_	Surface display

**Fig. 2:**
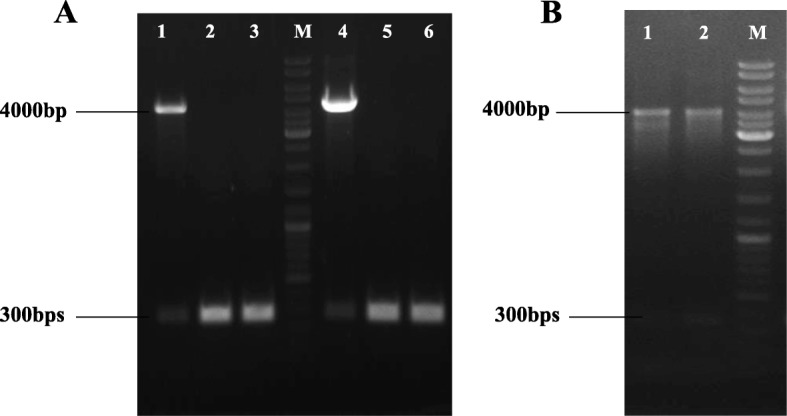
PCR verification of integrated plasmid in the *L. lactis* genome using attB flanking primers. **a** M) Generuler DNA ladder mix 1) integrated pS1nuc plasmid, 2) false positive clones, 3) negative control; 4) integrated pS2nuc plasmid, 5) false positive clone, 6) negative control. **b** 1) integrated pS3nuc, 2) integrated pS4nuc, M) Generuler DNA ladder mix

**Fig. 3:**
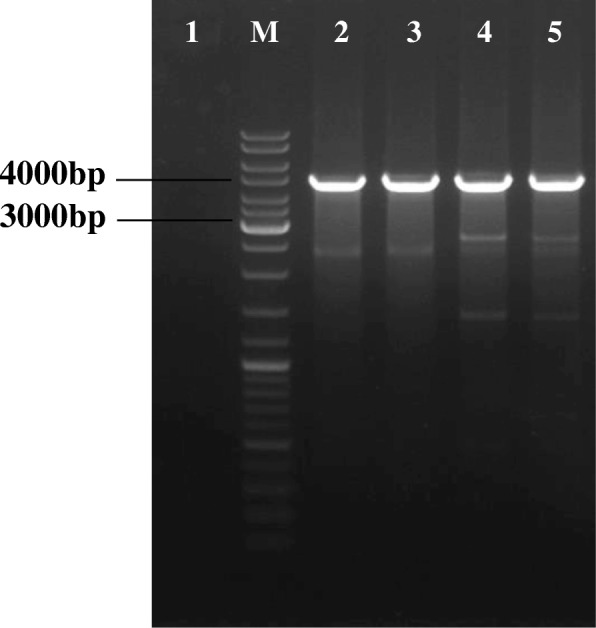
Gel electrophoresis of PCR verification using attB flanking primers of integrated surface displayed integrative plasmids in the *L. lactis* genome. M) Generuler DNA ladder mix, 1) negative control, 2) pSD1nuc, 3) pSD2nuc, 4) pSD3nuc, and 5) pSD4nuc

Figure 2 (a) and (b) show an example of PCR verification of selected integrants using primers flanking the attB site on the genome after 100 generations with the expected DNA size of 3944 bps, 3834 bps, 3927 bps and 3902 bps for pS1–4nuc, respectively, whereby the empty genomic DNA (negative control) was 300 bps. Figure 3 shows the gel electrophoresis image of the PCR amplified integrated surface displayed integrative plasmids (pSD1nuc, pSD2nuc, pSD3nuc, and pSD4nuc) with the expected DNA size of 4916 bps, 4875 bps, 4912 bps and 4943 bps respectively. However, some unspecific bands were also observed which is probably due to unspecific binding of the flanking primers in the genome

### Nuclease expression

**Fig. 4:**
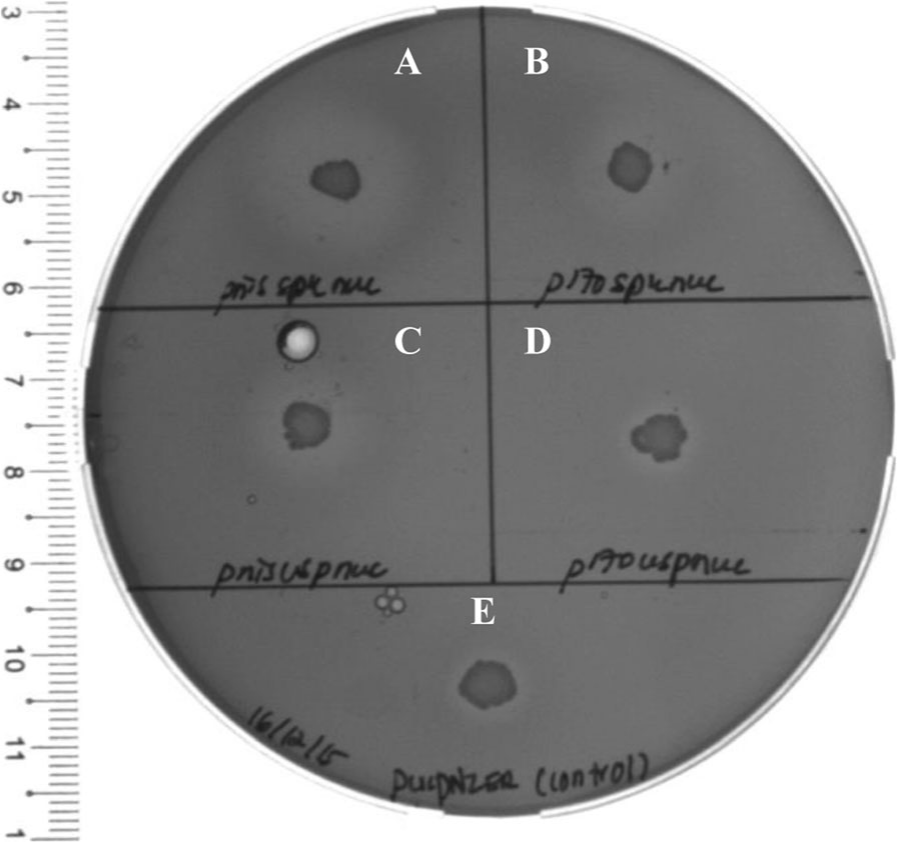
TBD test on integrated secretion plasmids clones, image taken using UV light gel documentation. **a**) pS4nuc, **b**) pS2nuc, **c**) pS3nuc, **d**) pS1nuc, and **e**) negative control

**Fig. 5:**
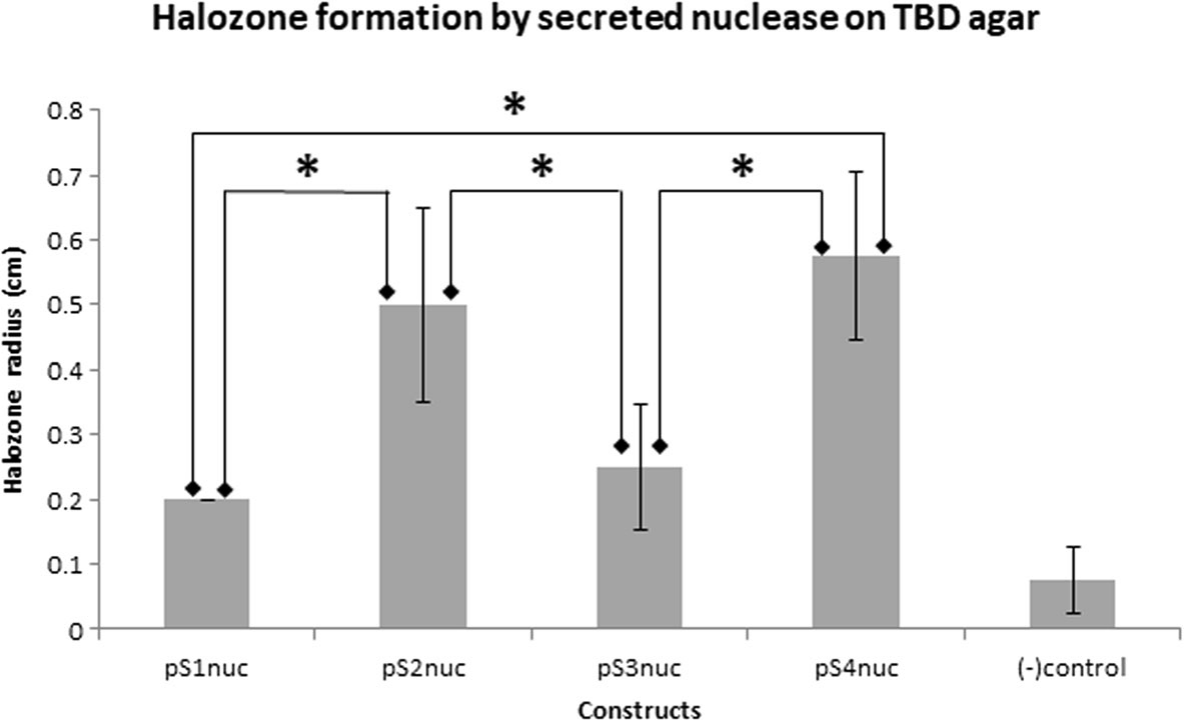
TBD assay of secreted nuclease by *L. lactis* harbouring integrated *nuc*. Data shown is an average of the radius of halozone of three replicates and *indicates significance *P* < 0.05. All samples are significant compared to the negative control

The secreted nuclease from the integrated secretion plasmids were determined by Toluidine Blue DNA (TBD) assay through colony overlay method as described by Lachica et al., (1971) [38]. The *L. lactis* strains harbouring integrated *nuc* were observed to form bright pink halo zone as compared to the negative control, a non-integrated *L. lactis*. Figure 4 shows the representation of halo zone formed around the bacteria culture on the TBD plate. The TBD assay concluded that *L. lactis* integrated with pS4nuc (driven by *P*_nisA_ promoter and SPK1 signal peptide) has the highest Nuc secretion with mean 0.575 cm followed by pS2nuc, pS3nuc and pS1nuc with mean 0.50 cm, 0.25 cm, 0.20 cm respectively (Fig. 5, raw data set in Additional file 1: Table S1). The ANOVA test indicates there is no significant difference in the expression of Nuc driven by *P*_170_ and *P*_nisA_ promoters. However, SPK1 showed better secretion of Nuc compared to USP45-LEISSTCDA fusion with significance of *P*-value < 0.05.

### Immunofluorescence analysis

**Fig. 6:**
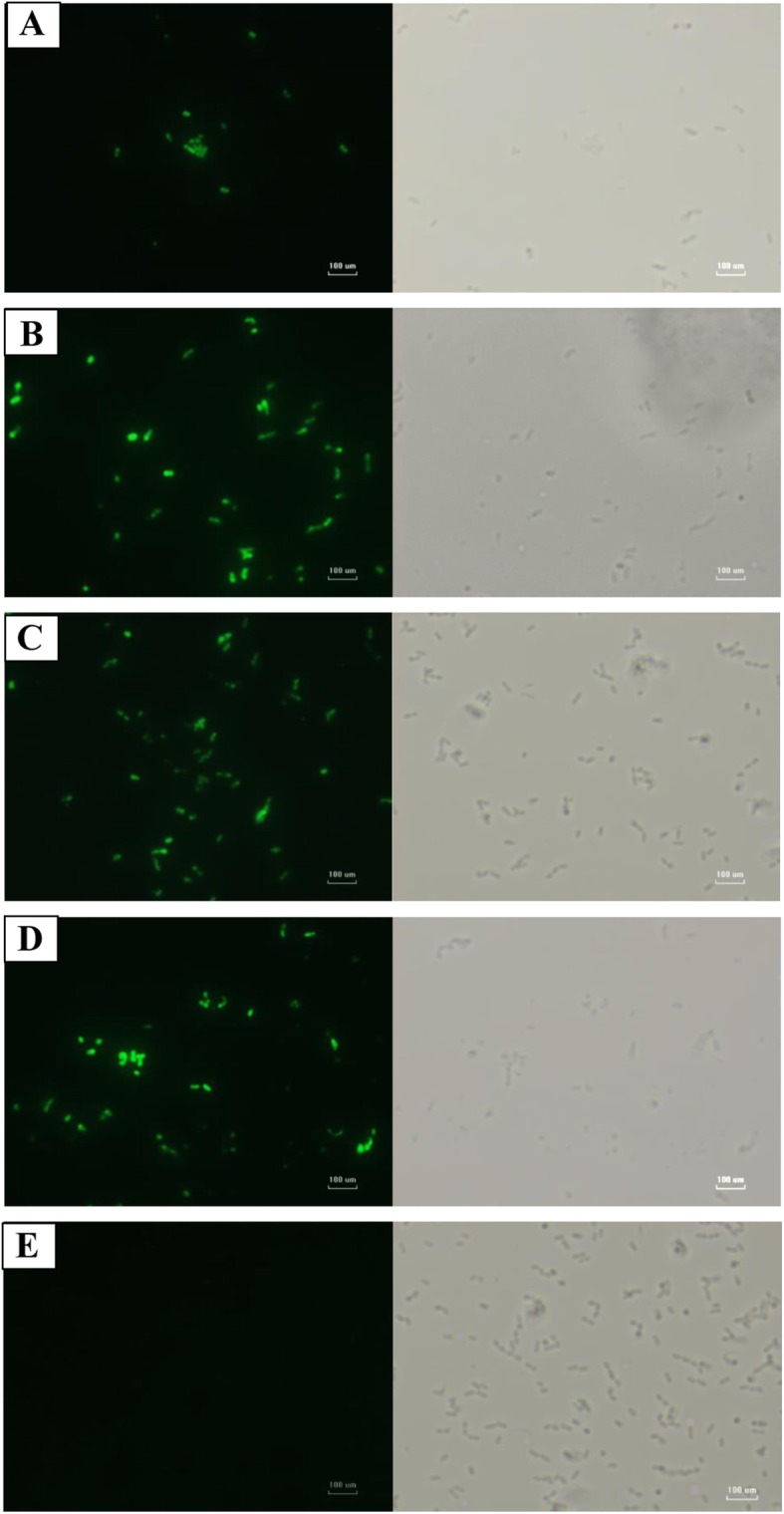
Fluorescent microscopy of *L. lactis* harbouring integrated pSDnuc1–4. The fluorescence images indicate surface anchored nuclease protein expressed from integrated plasmids; Left Panel: Green filter, Right Panel: Phase contrast; **a** pSD1nuc, **b** pSD2nuc **c** pSD3nuc, **d** pSD4nuc and non-integrated *L. lactis* NZ9000 as negative control (**e**)

The surface displayed nuclease on *L. lactis* NZ9000 was analysed to determine the bio-functionality of the cell wall anchoring motif, PrtP, an LPXTG anchoring system, via immunofluorescence microscope. It was observed that *L. lactis* integrated with pSDnuc1–4 were able to translocate and anchor the expressed Nuc onto the surface of the bacteria proven by the emission of green fluorescence signals as compared to the negative control, a non-integrated *L. lactis* (Fig. 6).

### Whole cell ELISA assay

**Fig. 7:**
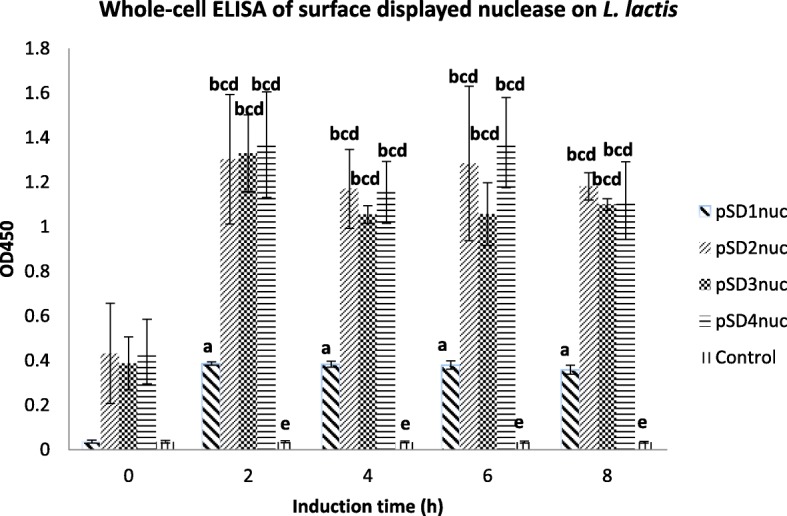
Whole-cell ELISA of induction time optimisation (from 0 to 8 h induction) for surfaced displayed nuclease on different integrated *L. lactis* constructs. ^abcde^ common letter on the bars mean no significance difference on the expression of nuclease by the respective integrative vectors (*P* < 0.05)

Quantitative expression of surface anchored nuclease was analysed by whole-cell ELISA assay with nuclease specific antibody. Among the surface displayed vector constructs, strain harbouring pSD1nuc showed extremely low signals, almost as low as the controls while pSD2-4nuc have almost the same signals intensities with no significance difference. Although it was observed that pSD1nuc and pSD3nuc strains driven by *P*_170_ and *P*_nisA_ promoter, respectively, show a highly significant difference with the latter showing greater signal intensities. We cannot conclude that *P*_nisA_ is a stronger promoter for surface displaying of proteins than *P*_170_ as this result was not consistent when comparing pSD2 and pSD4. Similarly, inconclusive results were also observed on the surface display of nuclease controlled by USP45 and SPK1 signal peptides as pSD1nuc and pSD2 showed significant intensity differences while pSD3 and pSD4 did not. Therefore, we can only conclude that the combination of *P*_170_ with USP45 was inferior compared to the other systems in the surface display integrative vectors (Fig. 7, raw data set in Additional file 2: Table S2).

## Discussion

In this work, two types of *Lactococcus lactis* integrative vectors utilizing the bacteriophage TP901–1 integrating system were successfully constructed. The TP901–1 is a temperate bacteriophage that resembles resolvase recombination mechanism. The TP901–1 attB was found to be located in a putative open reading frame encoded for competence protein, ComGC (Accession number: DJ61374.1) [39]. The integrative plasmid carrying the attP site recombines with the bacteria’s DNA sequence on the attB site, disrupting the open reading frame. However, up to date, there is no reports regarding the effect of this ORF disruption on the physiology of the cells [39, 40]. *Bacillus subtilis*, a naturally competent bacterium, utilises the proteins to take up of extracellular DNA into the cell. However, *L. lactis* and other dairy isolated bacteria were reported to be unable to take foreign DNA into the intracellular environment naturally. Thus, disruption of the protein might not have any significant effect.

The site-specific integrating system was designed to integrate the desired plasmid into *L. lactis* genome and express the gene of interest into the extracellular environment, where it is either fully secreted into the medium or surface displayed on *L. lactis* cell wall. The secretion integrative vectors, pS and the surface display integrative vectors, pSD were engineered with different combinations of promoters and signal peptides; *P*_170_ versus *P*_nisA_, and USP45 fused with LEISSTCDA versus SPK1 signal peptides alone, resulting in pS1, pS2, pS3 and pS4 secretion integrative vectors, and, pSD1, pSD2, pSD3 and pSD4 surface display integrative vectors, respectively (Table 2). Nuclease was used as a reporter to determine the ability of the bacteriophage integrating system to integrate into the *L. lactis* genome and the best combination of vector constructs to secrete or surface display the desired protein.

**Table 2:** Bacterial strains and plasmids used in this study

Strain or plasmid	Relevant phenotypes	References
Strains		
*E. coli* Top 10		Invitrogen
L. lactis NZ9000	MG1363 pepN::nisRK, host strain for all lactococcal plasmids	[41]
L. lactis pNZint	*L. lactis* harbouring pNZint plasmid	This study
Plasmid		
pCRTM-Blunt II-TOPO®	Zero Blunt® TOPO® PCR cloning Kit	Invitrogen
pUC19	2686 bps, Amp^R^, Cloning vector	New England Biolabs Inc. (NEB)
pNZ8048	3348 bps, Cm^R^, Nisin inducible expression vector	[41]
pMG36e	3611 bps, Erm^R^	[42]
pNZSPKNuc	Modified pNZ8048 carrying SPK1 signal peptide, *nuc* gene from *S. aureus*	Microbial Biotech Lab, UPM
pNZ170Gus	Modified pNZ8008, *P*_nisA_ was replaced with *P*_170_	Microbial Biotech Lab, UPM
pNZint	Modified pNZ8048 carrying integrase gene of TP901–1	This study
^a^pS1	Secretion integrative vector harboring *attP* of T901–1, *P*_170_, *E. coli* rep origin, USP45-LEISSTCDA, Em^R^	This study
^a^pS2	Secretion integrative vector harboring *attP* of T901–1, *P*_170_, *E. coli* rep origin, SPK1, Em^R^	This study
^a^pS3	Secretion integrative vector harboring *attP* of T901–1, *P*_nisA_, *E. coli* rep origin, USP45-LEISSTCDA, Em^R^	This study
^a^pS4	Secretion integrative vector harboring *attP* of T901–1, *P*_nisA_, *E. coli* rep origin, SPK1, Em^R^	This study
^a^pSD1	Surface display integrative vector harboring *attP* of T901–1, *P*_170_, *E. coli* rep origin, USP45-LEISSTCDA, PrtP anchor domain, Em^R^	This study
^a^pSD2	Surface display integrative vector harboring *attP* of T901–1, *P*_170_, *E. coli* rep origin, SPK1, PrtP_344_ anchor domain, Em^R^	This study
^a^pSD3	Surface display integrative vector harboring *attP* of T901–1, *P*_nisA_, *E. coli* rep origin, USP45-LEISSTCDA, PrtP_344_ anchor domain, Em^R^	This study
^a^pSD4	Surface display integrative vector harboring *attP* of T901–1, *P*_nisA_, *E. coli* rep origin, SPK1, PrtP_344_ anchor domain, Em^R^	This study

^a^ Plasmids had variations where nuclease reporters were cloned into the MCS and denoted with suufix-nuc in text

The data obtained showed that all the integrative vectors constructed were able to integrate into the genomic DNA of *L. lactis* and the integrants were stably integrated in the host genome. However, in this study, only PCR was used to verify integration of the plasmid. Further verification of the integration could possibly employ an alternative method which may include whole genome-sequencing to confirm that integration has taken place at the expected site and that ectopic integration has not taken place. However, it is unlikely that ectopic integration has taken place in the current study as the mechanism for integration using the attP/attB site is very specific, unlike homologous recombination where recombination may occur at different positions depending on the degree of similarity of the sequences.

The integrants constructed in this study were able to either secrete or surface display the reporter protein. The secreted Nuc results showed that pS2nuc and pS4nuc gave the highest secretion based on the radius of halo zone on TBD agar plates. Both pS2 and pS4 were driven by SPK1 signal peptide which demonstrated that the SPK1 signal peptide was superior over USP45 even though the USP45 was fused with synthetic propetide LEISSTCDA which was reported to enhance protein secretion [43]. This is despite USP45 being an endogenous signal peptide and the most commonly used signal peptide for secretion of heterologous protein in *L. lactis*. On the other hand, SPK1 is a signal peptide isolated from *Pediococcus pentasoceus* which was previously shown to possess comparative secretion efficiency to USP45 although total protein was at times much lower than in USP45 [44, 45].

From the analysis, there was no significant difference on the Nuc expression controlled by *P*_nisA_ and *P*_170_ promoters where the *P*-value for both pS1 against pS3 and pS2 against pS4 were above 0.05. Therefore, we can conclude that *P*_nisA_ and *P*_170_ promoters have comparable strength although the data showed a slight increase in the expression of Nuc by *P*_nisA_ promoter. The well-established NICE system with *P*_nisA_ is known for its strong and tightly regulated promoter [46–48] induced by nisin, whereas *P*_170_ is a lactococcal auto-inducible promoter by the accumulation of lactic acid in the culture medium which has the potential to be used in food-grade expression system [49–51]. In the industrial application where nisin induction will be costly for mass production and the safety concern of nisin in pharmaceutical applications made *P*_170_ promoter as a good alternative.

In the case of surface display integrative vectors, pSD, the integrated *nuc* was successfully expressed and anchored on the cell wall of *L. lactis* as shown in the immunofluorescence images (Fig. 6). The anchor domain PrtP_344_, which has the well-known LPXTG cell wall anchoring motif, successfully anchored the Nuc onto the surface of *L. lactis* [52–54]. Whole cell ELISA assay showed that pSD1nuc which had the combination of *P*_170_ promoter with USP45 signal peptide gave very low signals in the ELISA assay, although fluorescent signals were still detectable in immunofluorescence assay (Fig. 7). The expressed Nuc on the other clones which harboured pSD2nuc, pSD34nuc and pSD4nuc were comparable, although pSD2nuc and pSD4nuc, both which had the signal peptide SPK1 gave slightly better signals, although values were non-significant.

## Conclusions

In conclusion, the synthetic integrative vectors constructed in this study based on site-directed recombination via the attP/attB site were able to integrate the vectors into the lactococcal chromosome facilitated by integrase enzyme. The secretion integrative vectors, pS1, pS2, pS3 and pS4 were able to secrete Nuc protein into the extracellular region, while surface displayed integrative vectors; pSD1, pSD2, pSD3 and pSD4 were able to display Nuc reporter protein on the cell wall via the PrtP_344_ anchor domain. SPK1 signal peptide was shown to be superior over USP45 in secretion while the combination of *P*_170_ and USP45 was consistently inferior in both secretion and surface display integrative vectors. These constructed integrative expression vectors can be used to secrete and surface display recombinant protein and polypeptides stably for various applications, especially in live vaccine delivery.

## Methods

### Bacterial strains, plasmid and culture conditions

*Escherichia coli* Top 10 (Invitrogen, USA) cells were grown at 37 °C with agitation (250 rpm) in Luria Bertani (LB) broth or LB agar (Merck) (1.5% w/v bacteriological agar) with 50 μg/ml of kanamycin, 100 μg/ml of ampicillin or 75 μg/ml of erythromycin when necessary. *Lactococcus lactis* subsp. *cremoris* NZ9000 (MoBiTech GmbH, Germany) was grown in M17 (Merck) broth or agar supplemented with 0.5% (w/v) glucose as standing culture at 30 °C. Whenever required, erythromycin (10 μg/ml) and chloramphenicol (7.5 μg/ml) were used for the selection of recombinant *L. lactis* cultures. *E. coli* transformation was carried out using CaCl_2_ method [55] while *L. lactis* competent cell preparation and transformation were performed according to the protocol described by [56]. The electroporated cells were grown in SGM17 broth containing 0.5% (w/v) glucose and 0.5 M sucrose supplemented with chloramphenicol and/or erythromycin at a final concentration of 7.5 μg/ml and 10 μg/ml respectively and incubated at 30 °C for 2 days.

All plasmids used and developed in this study are listed in Table 2. *L. lactis* NZ9000 was used as the host for site-specific integration, providing the chromosomal attB site for the lactococcal phage TP901–1. NZ9000 was transformed in advanced with pNZint, which is a modified pNZ8048 harbouring the integrase gene (*int*), encoding the TP901–1 integrase (Genbank accession number: AF304433.1), to facilitate site-specific recombination. The constructed secretion integrative vectors pS1, pS2, pS3, pS4 and surface display integrative vectors pSD1, pSD2, pSD3, pSD4 are suicide vectors harbouring the *P*_170_ promoter or *P*_nisA_, USP45-LEISSTCDA or SPK1 signal peptide, MCS, TP901–1 attP site, *E. coli* origin of replication, erythromycin resistance gene (Em^R^). The integrative vectors were then transformed into the earlier *L. lactis* NZ9000 harbouring pNZint which is subsequently integrated into the attB site of the host genome.

### PCR amplifications and DNA manipulations

PCR amplification procedures were performed using either *Taq* DNA polymerase, for analytical purposes, or *Pfu* DNA polymerase, for cloning and sequencing (Fermentas, USA). The integrated DNA was verified via PCR with KOD FX Neo (TOYOBO, Japan). PCR primers were designed based on the known DNA sequences and relevant restriction enzymes (RE) were introduced via primers when needed (Table 3) which were synthesized by Integrated DNA Technologies (Singapore). DNA fragments were purified using GeneJET PCR purification Kit and GeneJET Gel Extraction Kit (Thermofisher, USA). Plasmids were isolated using Favorpep™ Plasmid DNA Extraction Mini Kit (Favorgen, Taiwan). Conventional and FastDigest RE and T4 DNA ligase were obtained from Thermofisher, USA.

**Table 3:** Primers used in this study

Primers	Sequences	RE
F-attB	CTACTGCTGCTTCACCAGTTT	–
R-attB	GTATGCAGCGATGTTGTTACCC	–
F-attP	GGAC **CTCGAG**TCCAACTCGCTTAATTGC	XhoI
R-attP	GGAC**GGATCC**GCTAAAACGTCTCAGAAA	BamHI
F-Int	GACG**CCATGG**CACATCATCATCATCATCATATGACTAAGAAAGTAGCAATCTATAC	NcoI
R-Int	GCAC**GAGCTC**TTAAGCGAGTTGGAATTTAAATATG	HindIII
F-PnisA	GCG**AGATCT**AGTCTTATAACTATAC	BglII
R-PnisA	GCA**GATATC**GTGAGTGCCTCCTTATAAT	EcoRV
F-P170	GCGAC**AGATCT**GAACTATGAATATC	BglII
R-P170	GCAC**GATATC**AACTGTTCTTTTTTAATTTTT	EcoRV
F-USP45	GACG**GATATC**ATGAAAAAAAAGA	EcoRV
R-USP45	CTGA**GTCGAC**GATATTTCGAGAGC	SalI
F-SpkNuc	GCGT**GATATC**ATGAAAAAAATATTAACGTTGGTAT	EcoRV
R-NucH	GGC**GAGCTC**TTAGTGGTGATGATGGTGA	SacI
F-NucEV	GCGT**GATATC**ATGAAAAAAATATTAACGTTGGTAT	EcoRV
F-NucNI	CGAG**CCATGG**CTATGAAAAAAATATTAACG	NcoI
R-NucEI	CGCG**GAATTC**TTGACCTGAATCAGCGTTGTCTTCG	EcoRI
F-Em	GACG**GGATCC**AATCAGGCTTGATCCCCAGTAAGTC	BamHI
R-Em	GACG**AGATCT**AGGATGAGGAGGCAGATTGCCTTG	BglII
F-PrtP344	GAGC**GAATTC**TGGCTCTAGAGGATCCAAGTC	EcoRI
R-PrtP344	TAAACTAT**GCGGCCGC**GACAGGCTATTCTTC	NotI
F-E. coli Ori	GGAG**GGATCC**GGCGTAATCATGGTCATAG	BamHI
R-E. coli Ori	AGGC**GGATCC**CTGTCAGACCAAGTTTACTC	BamHI

RE sites are in bold

### Plasmid constructions

The respective genes and DNA fragments were PCR amplified using primers listed in Table 3. The integrative vectors were constructed based on the pNZ8048 plasmid as backbone. A cassette consisting of USP45 fused with LEISSTCDA propeptide to increase secretion efficiency, pNZ8048 multiple cloning sites (MCS) modified to include more RE sites, pNZ8048 terminator (T), and TP901–1 bacteriophage attachment site (attP) was synthesized by Integrated DNA Technologies (Singapore) and denoted as attP cassette.

The vectors constructions strategy was divided into four phases; Phase I) Construction of PZatt cassette composed of the *P*_170_ promoter, USP45 signal peptide, LEISSTCDA propeptide and attP sequence; Phase II) Construction of the secretion integrative vectors (pS1–4); (a) Plasmid circularisation by incorporation of erythromycin resistant gene and *E. coli* replication origin into the PZatt cassette, denoted as pS1; (b) Replacement of *P*_170_ promoter with *P*_nisA_ promoter to produce pS3 plasmid; (c) Replacement of USP45-LEISSTCDA fusion with SPK1 signal peptide into each pS1 and pS3 plasmids to produce pS2 and pS4 plasmids (Additional file 3: Figure S1); Phase III) Construction of the surface display integrative vectors (pSD1–4) by incorporation of PrtP_344_ anchor domain into each secretion integrative vector; Phase IV) Cloning of reporter gene, nuclease (Additional file 4: Figure S2).

In Phase I, the attP cassette was ligated with *P*_170_ promoter to produce the PZatt cassette through EcoRV RE. In Phase II (a), the PZatt cassette was ligated with the erythromycin resistant gene (Em^R^) that was isolated from pMG36e to produce the PZER cassette using BglII RE. The PZER cassette was then fused with colE1 origin of replication from the pUC19 plasmid via BamHI RE to produce the functional circular plasmid, denoted as pS1. At each step of cassette construction, the cassettes were sub-cloned into pCRTM-Blunt II-TOPO® for maintenance. The *P*_170_ promoter of pS1 was cleaved out using BglII and EcoRV and was substituted with a similarly digested *P*_nisA_ promoter to produce pS3 plasmid (Phase II-b). On the other hand, the pS2 plasmid was constructed by replacing USP45 signal peptide with SPK1 using EcoRV and KpnI RE. To construct pS4 plasmid, USP45 in pS3 plasmid was cleaved out and was replaced with SPK1 signal peptide using NcoI and KpnI RE (Phase II-c). These resulted in secretion integrative plasmids (pS1–4) constructs.

The surface display integrative plasmids, pSD1–4 were constructed by insertion of PCR amplified PrtP_344_ into the respective secretion integrative vectors using EcoRI and NotI RE. All the successfully constructed integrative vectors were inserted with nuclease reporter gene, *nuc* in the MCS via NcoI and EcoRI sites to determine the functionality of the plasmids and to analyse the level of expression of each integrative vectors. Separately, the helper vector, pNZint (plasmid carrying integrase gene to assist integration) was constructed by cloning the integrase gene into pNZ8048 plasmid using NcoI and SacI RE.

### Cloning and integration of nuclease gene

The integration of foreign DNA into the host genome only happens with the presence of the integrase protein. Thus, *L. lactis* NZ9000 competent cells were first transformed with pNZint helper plasmid and the positive transformants were verified by restriction enzyme digestion and sequencing. The integration of the newly constructed integrative vectors harbouring the nuclease reporter was conducted in a few steps. First, *L. lactis* NZ9000 harbouring pNZint was made competent using protocol described by Holo and Nes (1989) with addition of 7.5 μg/ml of chloramphenicol into the culture media. Next, the competent cells were transformed with each integrative vector constructed according to the mentioned protocol with minor modifications. During the transformation process, after an hour of incubation, 20 ng/ml of nisin was added into the transformants to allow the expression of integrase protein and integration of the plasmid harbouring the nuclease gene. Transformants were then plated on SGM17 agar plate supplemented with 10 μg/ml of erythromycin and incubated at 30 °C for 2–3 days. The colonies were then verified with PCR using attB flanking primers (F-attB and R-attB) and were sent for sequencing. The positive colonies were used for further analysis.

### Stability tests

Doubling time of *L. lactis* was first determined using the *L. lactis* growth profile which was plotted prior. From the doubling time, cells were grown up to 100 generations by subculturing the cells to fresh media before it reaches stationary phase. After roughly 100 generations, cells were plated onto non-selective GM17 media. Then, colonies were transferred onto selective GM17 media containing 10 μg/ml of erythromycin due determine if the cells still possess antibiotic resistance. For further verification, genomic DNA was extracted from the integrants and verified using PCR with attB flanking primers to determine the presence of the integrated plasmid.

### Expression of heterologous protein

The expressed heterologous protein was determined by Western blotting. The grown culture of *L. lactis* harbouring integrated *nuc* was harvested. The secreted Nuc in the medium was harvested using 100% trichloroacetic acid, TCA (Merck, Germany) and the cell pellet was sonicated to harvest intracellular Nuc. Briefly, the harvested protein was added with loading buffer and boiled for 10 min. Treated samples were ran on SDS-polyacrylamide gel electrophoresis and then transferred to polyvinylidene difluoride (PVDF) membrane (ThermoFisher, USA). After blocking with 2% BSA at room temperature for 2 h, the membrane was incubated with the nuclease specific primary antibody, *Staphylococcus aureus* anti-nuclease antibody (LSBio, USA) at 1:10000 dilution followed by a secondary antibody goat anti-rabbit IgG H&L (HRP) (Abcam, USA) at 1:3000 dilution for another hour. The labelled protein on the membrane was detected using DAB substrate (Amresco, USA). On the other hand, the integrase protein was detected using 1:1000 dilution of primary antibody, Pierce™ 6X-His epitope tag antibody (ThermoFisher, USA) and 1:1000 dilution of secondary antibody, Peroxidase Conjugated Affinity Purified Goat anti-Mouse IgG (OriGene, USA).

### Nuclease assay (TBD assay)

Toluidine Blue DNA Agar was used for the detection of thermostable deoxyribonuclease activity. The agar was prepared according to the manufacturers protocol (HiMedia, India). Nuclease activity was detected using colony overlay as described by Lachica et al. (1970) [38] with minor modifications. *L. lactis* strain harboring *nuc* gene was spotted into the Brain Heart Infusion (BHI) agar (Oxoid, UK) and incubated overnight at 30 °C. Then, 15 ml of boiled TBD agar that has been cooled to 50 °C was poured onto the overnight cultured BHI agar. The overlaid agar was incubated at 37 °C for 4 h and bright pink halos observed.

### Immunofluorescence microscopy analysis

Cell wall anchoring protein was determined by immunofluorescence microscopy based on fluorescence conjugated secondary antibody tagging. The bacteria suspected to surface display nuclease were harvested, washed with PBS, applied to microscope slides, and fixed with 4% (v/v) formaldehyde. The cells were blocked with 3% (w/v) of BSA for 30 min at RT and washed thrice. The slides were incubated for 1 h with anti-nuclease antibody (diluted in 1:500 in PBS and 1% BSA) (LSBio, USA) on a slide chamber at RT, washed thrice with PBS. Then, the cells were labelled with polyclonal anti-nuclease primary antibody raised in rabbit (LSBio, USA) at 1:500 dilution and incubated for 1 h at RT. The slide was washed thrice with 1X PBS for 10 min and then incubated with anti-rabbit secondary antibody conjugated with Alexa Fluor 488 (Merck, USA) at 1:1000 dilution with 1 h incubation at RT followed by washing. The samples were analysed using Zeis Confocal Microscope (Carl Zeis, Germany) fixed with green filter.

### Whole cell ELISA assay

The anchored recombinant protein was quantified using ELISA assay with the specific antibody. The overnight cultures were transferred into fresh media and incubated at 30 °C until reached OD_600_ 0.5 and was induced with 20 ng/ml nisin. The cultures were harvested and normalized at constant OD_600._ They were then fixed with 4% (w/v) paraformaldehyde at RT, washed with 1X PBS and then blocked with 3% (w/v) BSA before washed again. The cells were then incubated with primary polyclonal nuclease anti-rabbit antibody (LSBio, USA), washed and then incubated with HRP conjugated secondary anti-rabbit antibody (Abcam, USA). The treated cells were transferred into 96 wells plate and incubated with substrate at RT in the dark. The reactions were stopped with 1 M H_2_SO_4_ (Sigma, USA)_._ The samples were then analysed using ELISA reader at OD_450_ (Magellan for F50, USA).

## Supplementary information

**Additional file 1:** **Table S1**. Data set of TBD assay of secreted nuclease.

**Additional file 2:** **Table S2.** Data set of whole-cell ELISA of surface displayed nuclease.

**Additional file 3:** **Figure S1.** Schematic representations of the construction strategy of the integrative vectors. Phase I) constructions of PZatt cassette backbone; Phase II) construction of secretion integrative vectors, (a) pS1 plasmid, (b) pS3 plasmid, (c) pS2 and pS4.

**Additional file 4:** **Figure S2.** Schematic representations of the construction strategy the integrative vectors. Phase III) construction of surface display integrative vectors, (pSD1–4); Phase IV) cloning of *nuc* gene into each integrative vectors.

## Data Availability

All data generated or analysed during this study are included in this published article (and its supplementary information files).

## Abbreviations

ANOVA: Analysis of variance
attB: Bacteria attachment site
attP: Phage attachment site
ELISA: Enzyme-linked immunosorbent assay
GRAS: Generally Recognised as Safe
LAB: Lactic Acid Bacteria
pS: Secretion integrative vector
pSD: Surface display integrative vector
RE: Restriction Enzyme
TBD: Toluidine Blue DNA
