# Supplementary figures and images for: Engineering integrative vectors based on phage site-specific recombination mechanism for Lactococcus lactis
Source: BMC Biotechnol. 2019 Nov 27;19:82. doi: 10.1186/s12896-019-0575-x (PMC6882331; doi:10.1186/s12896-019-0575-x)

**Phase I**

**Phase II (b)**


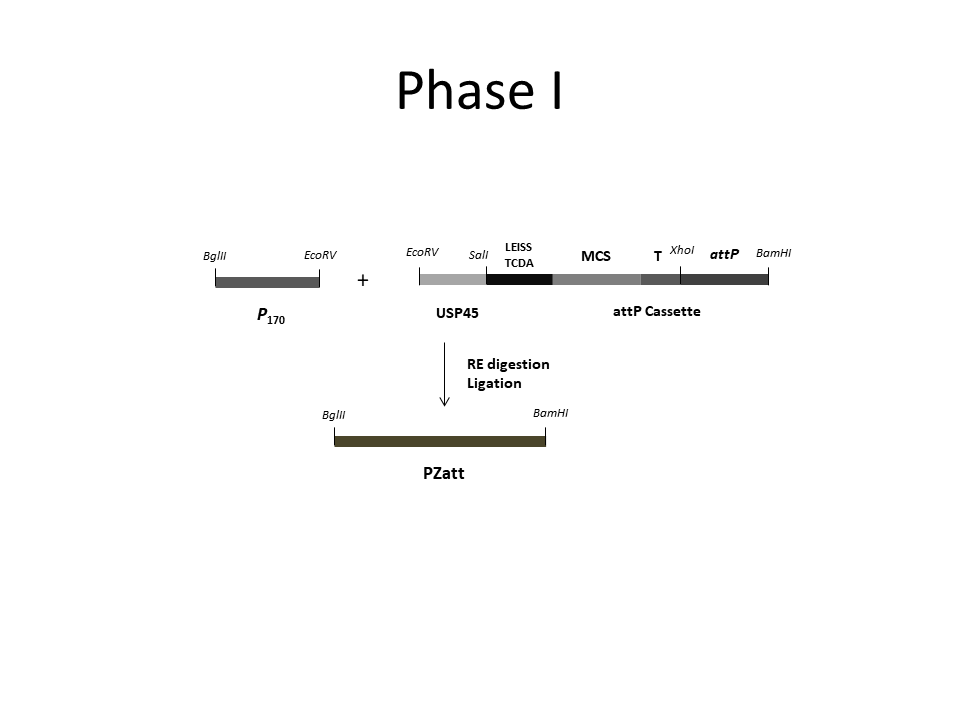


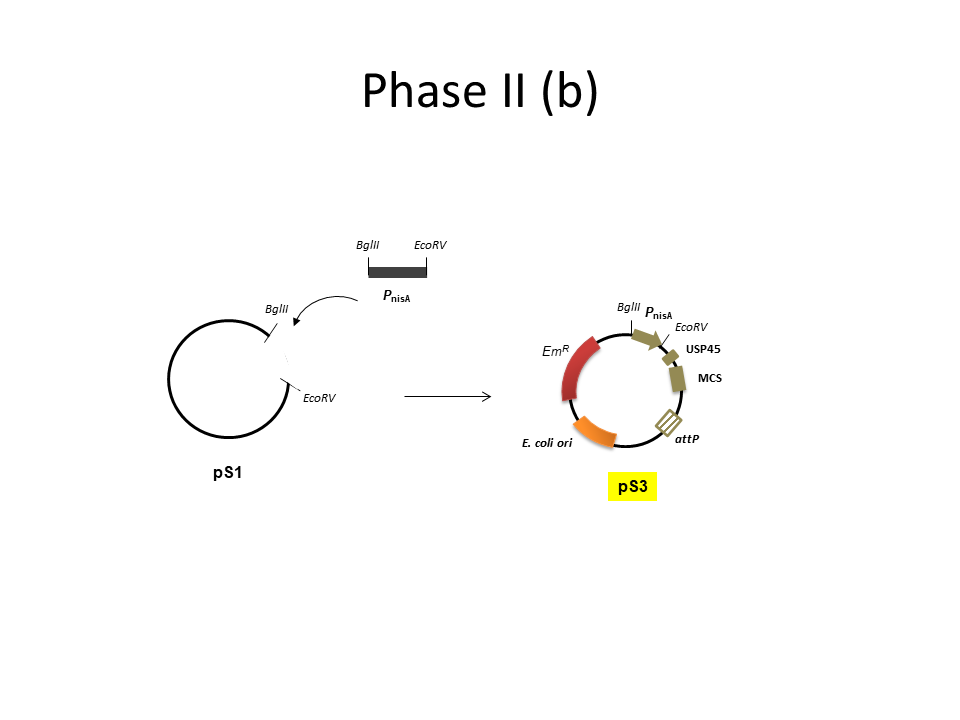


**Phase II (a)**


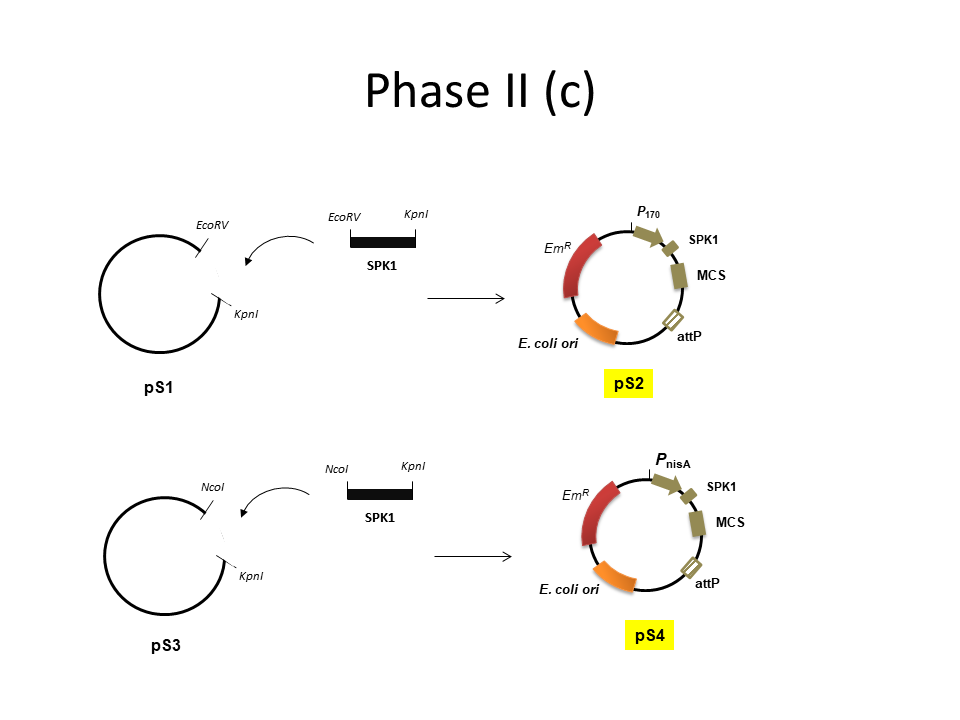


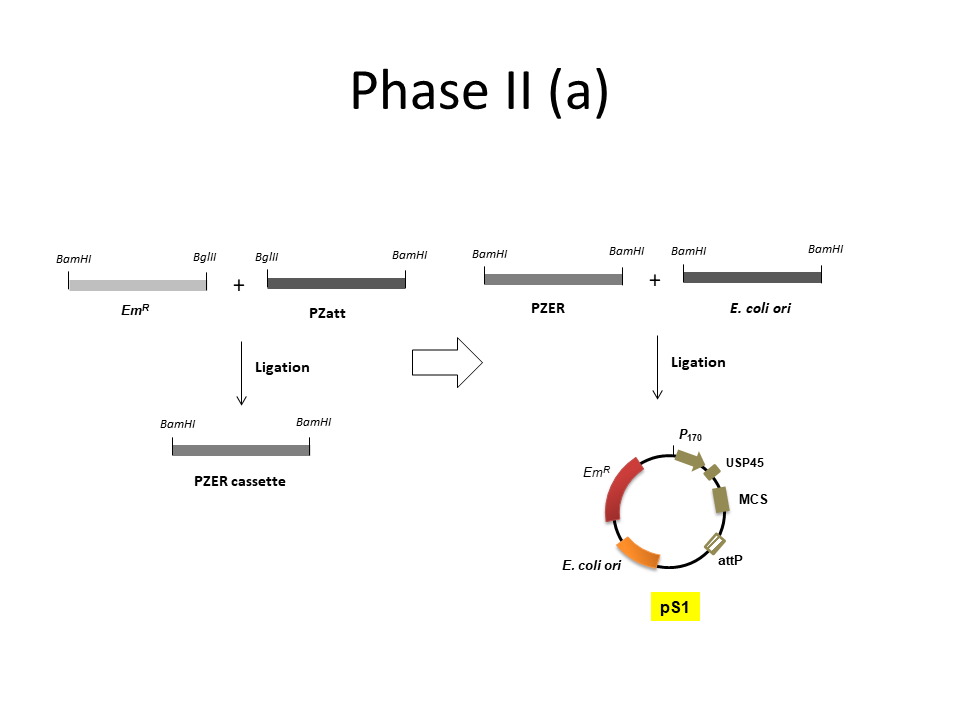


**Phase II (c)**

Supplement: Supplementary file 3 — Additional file 3: Figure S1. Schematic representations of the construction strategy of the integrative vectors. Phase I) constructions of PZatt cassette backbone; Phase II) construction of secretion integrative vectors, (a) pS1 plasmid, (b) pS3 plasmid, (c) pS2 and pS4. [file 12896_2019_575_MOESM3_ESM.docx]

**Phase III**


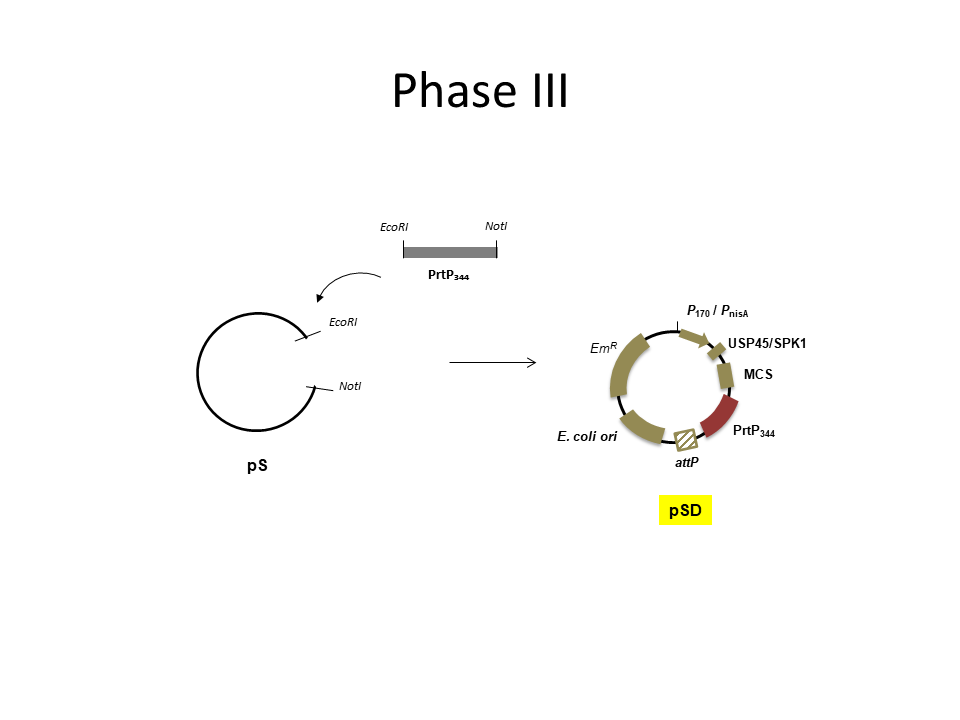


**Phase IV**


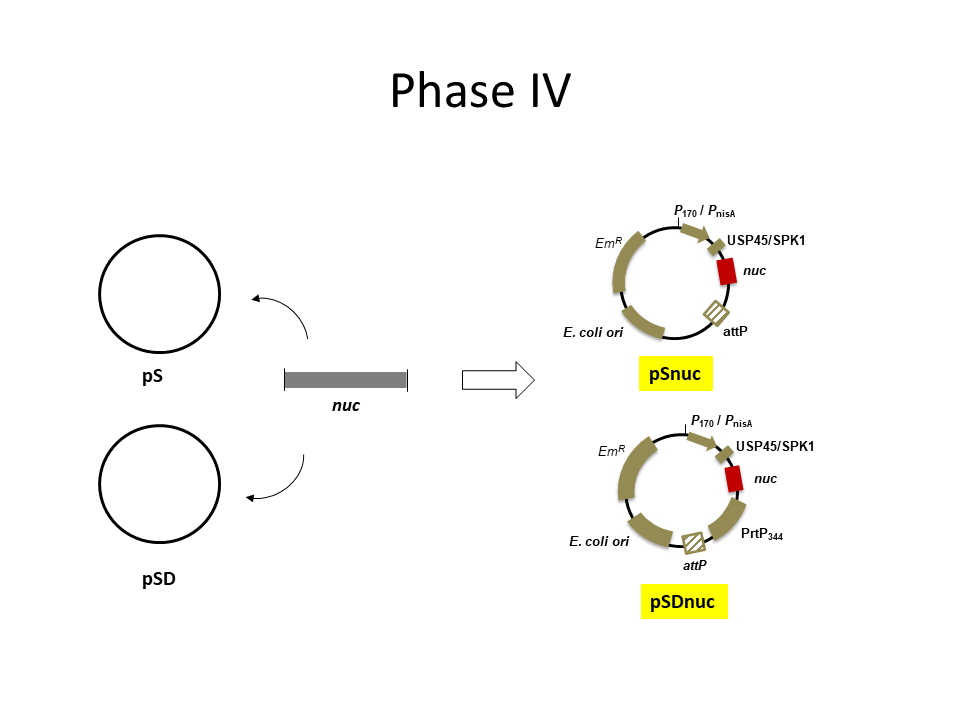

Supplement: Supplementary file 4 — Additional file 4: Figure S2. Schematic representations of the construction strategy the integrative vectors. Phase III) construction of surface display integrative vectors, (pSD1–4); Phase IV) cloning of nuc gene into each integrative vectors. [file 12896_2019_575_MOESM4_ESM.docx]
